# Cytosolic and Nuclear Co-localization of Betalain Biosynthetic Enzymes in Tobacco Suggests that Betalains Are Synthesized in the Cytoplasm and/or Nucleus of Betalainic Plant Cells

**DOI:** 10.3389/fpls.2017.00831

**Published:** 2017-05-18

**Authors:** Ning Chen, Zhi-Hai Yu, Xing-Guo Xiao

**Affiliations:** State Key Laboratory of Plant Physiology and Biochemistry, College of Biological Sciences, China Agricultural UniversityBeijing, China

**Keywords:** betalain biosynthesis, cDOPA5GT, CYP76AD1, cytoplasm, DODA1 (DOD), nucleus, subcellular localization, subcellular compartment

## Abstract

Betalains replace anthocyanins as color pigments in most families of Caryophyllales. Unlike anthocyanins, betalains are derived from tyrosine via three enzymatic steps: hydroxylation of L-tyrosine to L-3,4-dihydroxyphenylalanine (L-DOPA; step 1), and conversion of L-DOPA to betalamic acid (step 2), and to *cyclo*-DOPA (cDOPA; step 3). The principal enzymes responsible for these reactions have been elucidated at the molecular level, but their subcellular localizations have not been explored; hence, the intracellular compartments wherein betalains are biosynthesized remain unknown. Here, we report on the subcellular localization of these principal enzymes. Bioinformatic predictors and N- and C-terminal GFP tagging in transgenic tobacco, showed that *Beta vulgaris* CYP76AD1 which mediates both steps 1 and 3, DODA1 that catalyzes step 2, and CYP76AD6 which also mediates step 1, were similarly localized to the cytoplasm and nucleus (although the P450s were also weakly present in the endoplasmic reticulum). These two compartments were also the principal locations of *Mirabilis jalapa* cDOPA5GT. The cytoplasmic and nuclear co-localization of these key enzymes in tobacco suggests that betalains are biosynthesized in the cytoplasm and/or nucleus of betalain-containing plant cells. Elucidation of the subcellular compartmentation of betalain biosynthesis will facilitate the bioengineering of the betalain biosynthetic pathway in non-betalain-containing plants.

## Introduction

**FIGURE 1:**
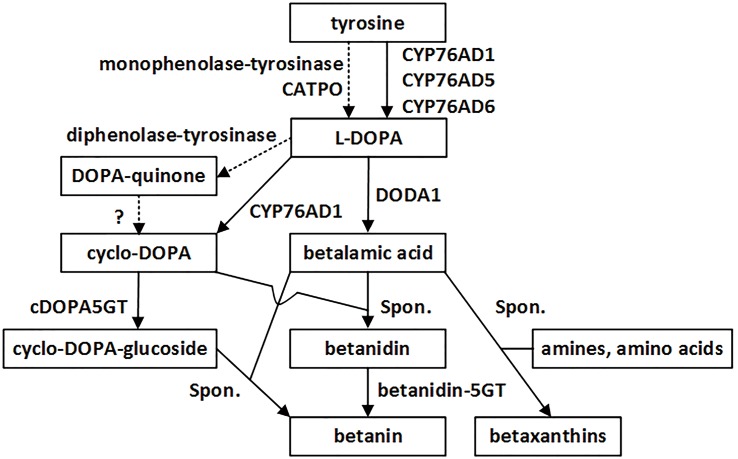
**The betalain biosynthetic pathway starting from tyrosine.** Two enzymes, CYP76AD1 and DODA1 mediate all three major reactions for formation of betalain backbone. Solid lines, reactions approved by experiments at biochemical and molecular levels. Dashed lines, reactions proposed in the literatures. Spon., spontaneous reactions.

Betalains are a group of water-soluble nitrogenous pigments, comprising yellow betaxanthins and red-violet betacyanins. They occur in most families of the order Caryophyllales, where they replace anthocyanins as major plant pigments. Their biosynthetic pathway is very simple, and essentially involves only three principal enzymatic steps (**Figure 1**; Strack et al., 2003; Han et al., 2009; Hatlestad et al., 2012; Gandia-Herrero and Garcia-Carmona, 2013; Schwinn, 2016): (1) 3′-hydroxylation of L-tyrosine to form L-3,4-dihydroxyphenylalanine (L-DOPA), (2) conversion of L-DOPA to betalamic acid (BA), the common chromophore of the yellow and red betalains, and (3) transformation of L-DOPA to *cyclo*-DOPA (cDOPA). Once BA is generated, it can spontaneously condense with amino acids or amines to form yellow betalains, the betaxanthins. It may also spontaneously condense with cDOPA to form the red betalain, the betanidin, which is then glycosylated to form diverse betacyanins (Vogt et al., 1999; Vogt, 2002).

With the arrival of “a golden age for betalain research” (Schwinn, 2016), we now know clearly that DOPA 4,5-dioxygenase (DODA1, DOD, DOD1) is responsible for the conversion of L-DOPA to BA (step 2; Girod and Zryd, 1991; Christinet et al., 2004), and that a newly discovered cytochrome P450, CYP76AD1, from beet (*Beta vulgaris*), is responsible for the transformation of L-DOPA into cDOPA (step 3; Hatlestad et al., 2012). The CYP76AD1 was discovered fortuitously in yeast, to able to perform catalysis of the hydroxylation of L-tyrosine to form L-DOPA (step 1) as a step in the possible production of pharmaceutical benzylisoquinoline alkaloids (DeLoache et al., 2015), and this role was subsequently confirmed *in planta* by Polturak et al. (2016). Furthermore, Polturak et al. (2016) showed that beet CYP76AD6, a member of paralogs of CYP76AD1, is also capable of the hydroxylation of L-tyrosine. This redundant role has also been attributed to beet CYP76AD5 (Sunnadeniya et al., 2016) and proposed for the monophenolase activity of the polyphenol oxidase (PPO)-type tyrosinase (Mueller et al., 1996; Steiner et al., 1996; Harris et al., 2012), as well as for the catalase–phenol oxidase (CATPO; Teng et al., 2016) in mushrooms and higher plants.

Given the recent rapid progress in the elucidation of the genes involved in the major steps of the betalain biosynthetic pathway, it is now possible to address the question of the cell compartment(s) in which the vacuole-localized betalains are synthesized. It is well known, a protein is transported to a specific subcellular localization to perform its function once synthesized in the cells (Zhang et al., 2007; Itzhak et al., 2016; Xiong et al., 2016). Therefore, determination of wherein the betalains are actually synthesized can be addressed by determining the subcellular localization of those enzymes responsible for the three major steps of the betalain biosynthetic pathway. DeLoache et al. (2015) reported that the C-terminal Venus-tagged CYP76AD1 appeared to be localized primarily to the endoplasmic reticulum (ER) in transgenic yeast cell, whereas the N-terminal RFP-fused DODA1 was localized to the cytoplasm. To the best of our knowledge, there is still no information on the subcellular localization of these enzymes in plants, though DODA1 has been predicted to be located in the cytoplasm (Christinet et al., 2004). In this paper, for the first time, we provide cell biological evidence in living plant cells that all the key enzymes required for the principal reactions of the betalain biosynthetic pathway are co-localized to the cytoplasm and the nucleus.

## Materials and Methods

### Plant and Gene Materials

Tobacco plants (*Nicotiana benthamiana*) were grown at 24 ± 2°C under a 16 h/8 h light/dark cycle in a culture room.

*Beta vulgaris* (*Bv*) *CYP76AD1* (GenBank accession HQ656023.1), *BvCYP76AD6* (accession KT962274), *BvDODA1* (accession HQ656027.1) and *Mirabilis jalapa* (*Mj*) *cDOPA5GT* (accession AB182643.1) were kindly provided by Dr. A. Aharoni (Weizmann Institute of Science, Rehovot, Israel). Binary vectors carrying 35S::N-GFP (Zhao et al., 2011), 35S::C-GFP (Zhao et al., 2015), the dual nuclear and cytoplasmic RFP marker, pGDR (Goodin et al., 2002), and the nucleolar RFP marker, pGDR-Fib (Wang et al., 2012), were the gifts from Drs. C-Q Sun, Y Guo, D-W Li and X-B Wang (China Agricultural University, Beijing, China), respectively. The nucleic acid stain DAPI (4′,6-diamidino-2-phenylindole) was purchased from Roche (Mannheim, Germany).

### Vector Construction

The full-length coding sequences of the above genes, with or without the stop codon, were PCR-amplified with gene-specific primers (**Table 1**) and linked to a pGEM-T vector (Promega, America). After sequencing verification, the coding sequences were inserted in-frame into 35S::N-GFP and 35S::C-GFP vectors using the corresponding restriction enzymes (**Table 1**), respectively, to generate 35S::GFP-gene and 35S::gene-GFP constructs.

**Table 1:** PCR primers used in this study.

Primer name	Sequence (5′–3′)	Restriction sites
GFPAD1-F	GGATCCATGGATCATGCAACATTAG	*Bam*H I
GFPAD1-R	GGTACCTCAATACCTAGGTATTGGAATAAGTTTTAAAGGCTTTGT	*Kpn* I
GFPAD6-F	GGATCCATGGATAACGCAACACTTGCTG	*Bam*H I
GFPAD6-R	GGTACCCTAGTTTCTGGGAAC	*Kpn* I
GFPBvDOD-F	GGATCCATGAAAATGATGAATGGTGAAG	*Bam*H I
GFPBvDOD-R	GTCGACCTAGGCTGAAGTGAACTTGTAGGAGCCATG	*Sal* I
GFP5GT-F	GGATCCATGACCGCCATTAAAATGAAC	*Bam*H I
GFP5GT-R	GGTACCTTATTGAAGAGAAGGTTCCAACTTAG	*Kpn* I
AD1GFP-F	GGTACCATGGATCATGCAACATTAG	*Kpn* I
AD1GFP-R	TCTAGAATACCTAGGTATTGGAATAAGTTTTAAAGGCTTTGT	*Xba* I
AD6GFP-F	GAGCTCATGGATAACGCAACACTTGCTG	*Sac* I
AD6GFP-R	TCTAGAGTTTCTGGGAACTGGAATAACTTGAAG	*Xba* I
BvDODGFP-F	GAGCTCATGAAAATGATGAATGGTGAAG	*Sac* I
BvDODGFP-R	TCTAGAGGCTGAAGTGAACTTGTAGGAGCCATG	*Xba* I
5GTGFP-F	GAGCTCATGACCGCCATTAAAATGAAC	*Sac* I
5GTGFP-R	TCTAGATTGAAGAGAAGGTTCCAACTTAG	*Xba* I
BvcDODGFP-F	GAGCTCATGAAAATGATGAATGGTGAAG	*Sac* I
BvcDODGFP-R	TCTAGAGGCTGAAGTGAACTTGTAGGAGCCGTG	*Xba* I
AcDODGFP-F	GAGCTCATGGGTAGTCAAGAAATCAT	*Sac* I
AcDODGFP-R	CCCGGGGCTTGAAACAAATTTG	*Sma* I


### Plant Transformation, and Confocal Observations

All GFP constructs, the dual nuclear and cytoplasmic RFP marker (nuc/cyt RFP marker) pGDR, and the nucleolar RFP marker pGDR-Fib, were transformed into *Agrobacterium tumefaciens* strain GV3101 and the resultant bacterial suspensions were infiltrated into young leaves of tobacco plants (*N. benthamiana*), following the method of Sparkes et al. (2006) as previously reported (Han et al., 2013). For co-infiltration, the bacterial suspension harboring the RFP marker, and that carrying the fused GFP construct, were mixed in a 1:1 ratio, and the resulting mixture was agro-infiltrated into the young leaves as described above. Two to three days post infiltration, the abaxial epidermis of the leaves was subjected to Carl Zeiss 710 confocal laser scanning microscopy (CLSM). The GFP and RFP channels were acquired by simultaneous scanning at excitation wavelengths of 488 and 568 nm, and the fluorescence signals were detected using an emission bandwidth of 505–530 nm for GFP and 580–630 nm for RFP. Chlorophyll autofluorescence was observed with an excitation wavelength of 488 nm and an emission bandwidth of 650–700 nm. To verify the nucleolar RFP as a nuclear marker, the leaves infiltrated with pGDR-Fib were stained with DAPI for 5 min, according to the manufacturer’s instructions, and then the abaxial epidermis of the leaves was subjected to CLSM (Leica SP5). The DAPI and RFP channels were acquired by simultaneous scanning at excitation wavelengths of 405 and 568 nm, and the fluorescence signals were detected using an emission bandwidth of 480–500 nm for DAPI and 580–630 nm for RFP. The images were first processed in the Zeiss LSM Image Browser software, and the combined images were generated using Photoshop.

### Database Searches and Bioinformatics Predictions

For database searches and bioinformatics predictions of chloroplast transit peptides, signal peptides and subcellular localizations, whole amino acid sequences of the four target enzymes were analyzed using the on-line signal peptide prediction programs TargetP1.1^1^, ChloroP1.1^2^, SignalP4.1^3^, and PredSL^4^, and the subcellular localization prediction programs WoLF PSORT II^5^, ProtComp 9.0^6^ and UniProtKB^7^. For all programs, the defaults “cut-off” and “Plant,” or “Eukaryotes” if “Plant” was not available, were selected.

## Results

### Prediction of Signal Peptides and Subcellular Localizations

Prior to the experimental work, we used a variety of on-line bioinformatics resources, both individual predictor and then integrator programs, to predict signal peptides, especially the chloroplast transit peptide (cTP), and the subcellular localizations of CYP76AD1 (AD1), CYP76AD6 (AD6), DODA1, and cDOPA5GT (5GT).

Neither a cTP nor a chloroplast localization was predicted for AD1, DODA1, or 5GT by any of the seven prediction programs used, although according to WolF PSORT and ProtComp, respectively, a low probability of chloroplast localization for AD1 and a very low one for 5GT could not be ruled out (**Table 2** and Supplementary Table S1). For AD6, a cTP was found by ChloroP, though not by SignalP or TargetP, and a chloroplast localization was predicted by WolF PSORT and PresdSL, though not by UniProt and ProtComp (**Table 2** and Supplementary Table S1).

**Table 2:** Comparison of signal peptides and subcellular localizations of CYP76AD1, CYP76AD6, DODA1, and cDOPA5GT predicted, N- and C-terminal GFP tagged.

		Cell localization (this study)		Cell localization- bioinformatic prediction								Signal peptide			
				
Gene name	Length (AA)	N-TERM	C-TERM	WoLF PSORT II _Over 2	ProtComp 9.0_Over 1				Uni Prot	PredSL		TargetP	SignalP		ChloroP
								
						PLDB	NN	ALL	LOC	LOC	CS	LOC	POS	SIG	cTP
AD1	497	NUC/CYTO/CHL	NUC/CYTO_ER	CYTO	PM	2.5	0.97	5.25	MEM	SP	25	SP	1–25	Y	N
				CHL	ER	2.5	0	4.6							
				ER											
AD6	499	NUC/CYTO/CHL	NUC/CYTO_ER	CHL	PM	2.4	1	5.2	MEM	CHL	8	SP	1–29	N	Y
				CYTO	ER	2.6	0	4.6							
DODA1	275	NUC/CYTO/CHL	NUC/CYTO	CYTO	CYTO	5.0	0	7.08	CYTO	O	U	O	1–70	N	N
				CYSK	CHL	0	0	1.48							
5GT	500	NUC/CYTO/CHL	NUC/CYTO/CHL	CYTO	EC	0	0.94	2.31	U	O	U	O	1–110	N	N
				CYTO_ER	MIT	0	0	1.03							
				NUC	ER	0	0	1.81							
				CYSK	PEROX	0	0.94	2.07							
					CHL	0	0	2.28							


*AD1, CYP76AD1; AD6, CYP76AD6; 5GT, cDOPA5GT; AA, amino acids; CHL, chloroplast; CYSK, cytoskeleton; CYTO, cytoplasm; CYTO_ER, both cytoplasm and endoplasmic reticulum; EC, extra cellular; ER, endoplasmic reticulum; MEM, membrane; MIT, mitochondria; NUC, nucleus; PEROX, peroxisome; PM, plasma membrane; C-TERM, C-terminal-tagged GFP; N-TERM, N-terminal-tagged GFP; PLDB, predicted location from database; NN, neural networks; ALL, combined results from LDB, PLDB, NN and PT; LOC, prediction of location; CS, cleavage site; U, unknown; O, any other location (expect cTP, mTP, and SP); SP, secretory pathway; POS, position; SIG, signal peptide; Y, yes; N, no; cTP, chloroplast transit peptide; mTP, mitochondrial targeting peptide.*

None of the four proteins was predicted to be localized to the nucleus, though a very low probability existed in the case of 5GT, whereas all the integrated prediction programs, except for PredSL, forecast a cytoplasm or plasma membrane localization for DODA1 and AD1 (**Table 2** and Supplementary Table S1). PredSL gave no information on the subcellular localization of DODA1, and it predicted AD1 as simply a secretory protein. For 5GT, the strongest prediction of localization was to the cytoplasm and to the ER, as indicated by WolF PSORT; in contrast, the other programs gave either no information or produced variable predictions, though there was little or no indication of localization to the chloroplasts.

From the above predictions, we may posit that AD1, DODA1, and 5GT are most likely localized to the cytoplasm (ER and plasma membrane included), but not to the nucleus or to the chloroplasts, whereas AD6 may be localized both to the chloroplast and to the cytoplasm.

With these prediction results in mind, we began our experiments using the GFP tag. Before proceeding, however, we verified, using the Leica sp5 CLSM, that the red fluorescence signal in the abaxial epidermal cells of the pGDR-Fib-transgenic tobacco leaves was indeed detected only in the nucleolus, and that it was localized in the center of a blue nucleus stained by DAPI (Supplementary Figure S1). With this confirmed, we went ahead and used the nucleolar marker RFP (pGDR-Fib) as the nuclear marker in the GFP tag experiments that followed.

### GFP Tagged at the N-terminus of the Gene of Interest (GFP-Gene)

DeLoache et al. (2015) showed that DODA1 with the RFP tag at the N-terminus (RFP-DODA1) was functional in yeast cells. Hence, it was plausible that N-GFP-fused DODA1 and N-GFP fusions of the other enzymes of the pathway should also be functional in plant cells. On this premise, we initiated the determination of the subcellular localization of AD1, AD6, DODA1, and 5GT by tagging GFP to their N-termini.

**FIGURE 2:**
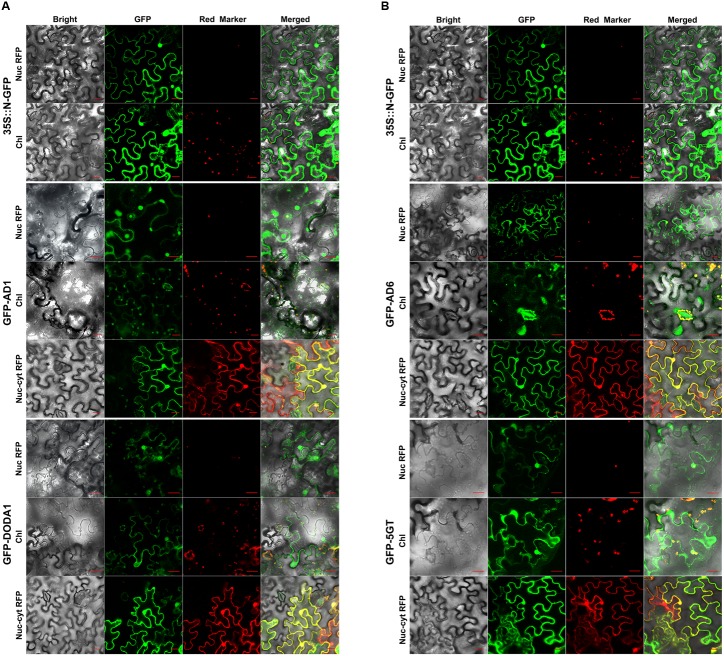
**Confocal GFP fluorescence scanning of N-terminal tagged gene transgenic tobacco leaf cells.** **(A)** GFP-CYP76AD1 and GFP-DODA1; **(B)** GFP-CYP76AD6 and GFP-cDOPA5GT. Every GFP construct and nucleolus-localized RFP marker or dual nucleus and cytoplasm RFP marker were co-transformed to tobacco (*N. benthamiana*) leaf epidermal cells by agro-infiltration, and 2 or 3 days post infiltration, the cells were assessed for fluorescence under Carl Zeiss 710 confocal laser scanning microscopy. For each enzyme, the upper panel shows red fluorescence from nucleolus marker RFP, the middle one, red fluorescence from chloroplast autofluorescence, and the lower one, dual nucleus and cytoplasm marker RFP. AD1, CYP76AD1; AD6, CYP76AD6; 5GT, cDOPA5GT; Nuc RFP, Nucleolus-localized RFP marker; Chl, Chloroplast autofluorescence; Nuc-cyt RFP, dual nucleus and cytoplasm RFP marker; GFP-Gene, GFP tagged at the N-terminus of the gene; Bar = 20 μm.

We first investigated AD1 (CYP76AD1), because it mediates two of the three main enzymatic steps that convert tyrosine to the betalain pigment backbone (as described above). We spliced GFP to the N-terminal of AD1 and expressed the fusion protein under the control of the constitutive CaMV 35S promoter (35S) in tobacco (*N. benthamiana*) epidermal leaf cells, with the 35S::N-GFP, the nuc/cyt RFP marker, and the nucleolus-localized RFP marker serving as controls. The transient expression of the construct demonstrated that the GFP-AD1 fusion protein was localized not only to the cytoplasm and nucleus but also to the chloroplasts, specifically to the stomatal guard cell chloroplasts (hereafter “guard cell chloroplasts”), as indicated by the overlay of the GFP fluorescence with the red chlorophyll autofluorescence (GFP-AD1 in **Figure 2A**). When co-expressed with the nucleolar marker RFP, the GFP-AD1 was co-localized with the marker to the nucleus, whereas when it was co-expressed with the nuc/cyt marker RFP, the green fluorescence overlapped with the red fluorescence in both the cytoplasm and the nucleus (GFP-AD1 in **Figure 2A**). For the free GFP (35S::N-GFP), the green fluorescence signal was detected in the cytoplasm and nucleus, but not in the free chloroplasts (i.e., non-guard-cell chloroplasts), nor in the guard cell chloroplasts (35S::N-GFP in **Figure 2A**).

Next, we studied the subcellular localization of DODA1 (DOD), the enzyme that catalyzes the conversion of L-DOPA to BA. The GFP signal of GFP-DODA1 was recorded in the cytoplasm, nucleus, free chloroplasts, and guard cell chloroplasts, and its green fluorescence in the chloroplasts overlapped with the red autofluorescence originating from the chloroplasts themselves (GFP-DODA1 in **Figure 2A**). The GFP signals occurring in the cytoplasm and nucleus were confirmed by the red signals from the nucleolar and nuc/cyt marker RFPs, respectively, when the GFP-DODA1 was co-expressed with the markers (GFP-DODA1 in **Figure 2A**).

After determining the subcellular compartmentalization of the two key enzymes responsible for the three principal steps of the pathway (**Figure 1**), we investigated whether AD6 (CYP76AD6), one of numerous paralogs of AD1 (CYP76AD1) in beet, showed the same subcellular localization as AD1. Like AD1, AD6 has been reported to catalyze tyrosine hydroxylation (step 1) (Polturak et al., 2016; Sunnadeniya et al., 2016). In the transiently transgenic tobacco epidermal leaf cells, the GFP signal of the GFP-AD6 fusion protein was clearly present in the cytoplasm, the nucleus, the guard cell chloroplasts and to some extent in the free chloroplasts; and this signal coincided with that of the chloroplast autofluorescence in the chloroplasts (including the guard cell chloroplasts), with the nucleolar RFP signal in the nucleus, and with the nuc/cyt RFP signal in the nucleus and cytoplasm (GFP-AD6 in **Figure 2B**).

Furthermore, we determined the intracellular localization of *Mj*cDOPA5GT (5GT). This 5GT was reported to catalyze the 5-*O*-glucosylation of *cyclo*-DOPA (Sasaki et al., 2005), and the glucosylated *cyclo*-DOPA has been shown to condense directly with BA to form betanin, one of the betacyanins, in transgenic tobacco leaves (Polturak et al., 2016). Similar to the findings above, the GFP-5GT fusion protein was found to be localized to the cytoplasm, the nucleus, the guard cell chloroplasts, and free chloroplasts; and its chloroplast-, nuclear-, and cytoplasmic-localization GFP signals were overlaid, respectively, with the red signals from the chloroplast autofluorescence, nucleolar marker RFP, and nuc/cyt marker RFP (GFP-5GT in **Figure 2B**).

The above results indicated that when fused with GFP at the N-terminus, the AD1, AD6, DODA1, and 5GT were targeted to almost the same subcellular compartments: nucleus, cytoplasm, and chloroplasts, including guard cell chloroplasts. These results disagreed with our bioinformatics-based predictions, except in respect of the cytoplasmic localization of the four proteins and the possible chloroplast localization of AD6. This divergence led us to fuse the GFP at another terminus, the C-terminus of the above four proteins, for the purpose of “arbitration.”

### GFP Tagged at the C-terminus of the Gene of Interest (Gene-GFP)

We successfully fused the GFP to the C-terminus of AD1, AD6, DODA1, and 5GT and identically expressed the fusion proteins under the control of the 35S promoter in tobacco epidermal leaf cells, with 35S::C-GFP, the nuc/cyt RFP marker (pGDR), and the nucleolar RFP marker (pGDR-Fib) serving as controls.

**FIGURE 3:**
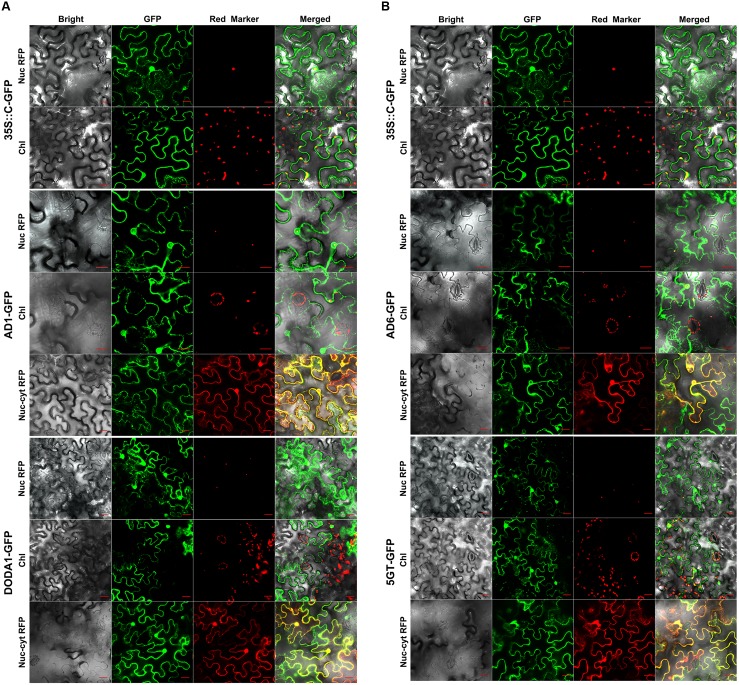
**Confocal GFP fluorescence scanning of C-terminal tagged gene transgenic tobacco leaf cells.** **(A)** CYP76AD1-GFP and DODA1-GFP; **(B)** CYP76AD6-GFP and -cDOPA5GT-GFP. The figure explanation is the same as that for **Figure 2**, except Gene-GFP, GFP tagged at the C-terminus of the gene.

In the transiently transgenic tobacco leaf cells, the C-terminal GFP-tagged CYP76AD1 fusion protein (AD1-GFP) was localized to both the nucleus, with a stronger green fluorescent signal in the nuclear membrane, and the cytoplasm, with a clear signal at the ER, but not to the free chloroplasts, nor to the guard cell chloroplasts and stoma (AD1-GFP in **Figure 3A**). These dual localization signals coincided with those of the nuc/cyt marker RFP, and the nuclear signal matched that of the nucleolar marker RFP when the fusion protein was co-expressed with the markers (AD1-GFP in **Figure 3A**). Similarly, the DODA1-GFP fusion protein was localized only in the cytoplasm and nucleus, and the fluorescence signal in the nucleus was more evenly distributed than that of AD1-GFP (DODA1-GFP in **Figure 3A**). For the C-GFP-tagged AD6, the fusion protein shared the same subcellular compartments as AD1-GFP, being present in both the nucleus and the cytoplasm, with a pronounced signal at the ER, but not in the chloroplasts or in the stoma (AD6-GFP in **Figure 3B**). Furthermore, the GFP signals of the nucleus/cytoplasm-localized DODA1-GFP and AD6-GFP overlapped with the RFP signals of the nuc/cyt marker and with those of the nucleolar marker (DODA1-GFP and AD6-GFP in **Figure 3**). Regarding 5GT-GFP, the fusion protein was clearly present in the nucleus, cytoplasm, free chloroplasts, and guard cell chloroplasts (5GT-GFP in **Figure 3B**). Its green signals in the chloroplasts, nucleus, and cytoplasm coincided with the red signals from the chloroplast auto-fluorescence, nuc/cyt marker RFP, and nucleolar marker RFP, respectively, albeit the auto-fluorescence signal was much weaker (5GT-GFP in **Figure 3B**).

In summary, the above C-GFP-tag results demonstrated that AD1, AD6, and DODA1, when tagged with GFP at their C-terminal, were localized to the cytoplasm (ER included) and to the nucleus, but not to free chloroplasts or guard cell chloroplasts, nor to the stoma. By contrast, the C-GFP-tagged 5GT was present in the chloroplasts, including the guard cell chloroplasts, in addition to its cytoplasmic and nuclear locations.

## Discussion

The subcellular localization of a protein is often tied to its function (Grefen et al., 2008; Bononi and Pinton, 2015). In the betalain biosynthetic pathway, BvCYP76AD1 (AD1), BvDODA1 (DOD), and BvCYP76AD6 (AD6) have already been verified at the molecular level as the major enzymes involved (**Figure 1**; Christinet et al., 2004; Hatlestad et al., 2012; DeLoache et al., 2015; Polturak et al., 2016), and MjcDOPA5GT (5GT) is responsible for the direct formation of betanin by glucosylation of *cyclo*-DOPA prior to condensation with BA (Sasaki et al., 2005; Polturak et al., 2016). The subcellular localizations of these enzymes are very likely indicative of the sites of their catalytic function and, therefore, of the intracellular compartments in which the betalains (and their intermediates) are biosynthesized. On the basis of this reasoning, we investigated the subcellular localization of these four enzymes (CYP76AD1, CYP76AD6, DODA1, and cDOPA5GT).

We started this work by using an on-line bioinformatics prediction suite comprising three “individual predictors” and four “integrators” (Supplementary Table S1), according to the suggestions of Tanz and Small (2011). We then used GFP as a tag at the N-terminal and then the C-terminal of the proteins, because of its demonstrated advantage over other plant subcellular localization methods, e.g., the immunohistochemical method (Goodin et al., 2002; Nelson et al., 2007; Geldner et al., 2009; Tanz et al., 2013).

Our results showed that the predictors and the N- and C-terminal GFP tagging all pointed to the cytoplasmic localization of DODA1, followed by the cytoplasmic localization of AD1 and then that of 5GT (**Figures 2**, **3**, **Table 2**, and Supplementary Table S1). However, the greatest level of disagreement related to the possible chloroplast localization of the four proteins (**Table 2**). It is well known that most chloroplast proteins encoded by nuclear genes have N-terminal targeting sequences, i.e., cTPs (von Heijne et al., 1989; Bruce, 2000). Among the four proteins studied here, only AD6 was predicted for the presence of a cTP by ChloroP and chloroplast-localized by PredSL and Psor (**Table 2** and Supplementary Table S1). However, the N-terminal GFP tagging showed that all four proteins were localized to the chloroplasts, including guard cell chloroplasts (GFP-Gene in **Figure 2** and **Table 2**). The potential for discrepancies among the predictors, and between predicted and experimental results, were noted in the development of multicolored organelle markers for co-localization studies in *Arabidopsis* and other plants (Nelson et al., 2007).

We observed that results of the C-terminal tagging were not always in concordance with those of the N-terminal tagging. For 5GT, the N- and C-terminal GFP tagging were in accord concerning its intracellular localization (GFP-5GT in **Figure 2B**; 5GT-GFP in **Figure 3B**), but they deviated over the chloroplast localization for AD6, AD1, and DODA1: the C-terminal tagging localized AD6, AD1, and DODA1 to the nucleus and cytoplasm, whereas the N-terminal tagging placed them not only in the nucleus and the cytoplasm but in the chloroplasts as well (GFP-Gene in **Figure 2** vs. Gene-GFP in **Figure 3** and **Table 2**). The C-terminal tagging of BvDODA1 was supported by both the C-terminal GFP-tagged DODA1 from red Swiss chard (accession no. KU644145) and the DODA1 from red amaranth (accession no. KU644143) (Supplementary Figure S2). In addition, the GFP signal in the nucleus of GFP-AD1 was evenly distributed, whereas that in the nucleus of AD1-GFP was more pronounced in the nuclear membrane (GFP-AD1 in **Figure 2A** vs. AD1-GFP in **Figure 3A**). Furthermore, the fluorescence signals of AD1-GFP and AD6-GFP were more manifest in the ER of the cytoplasm than those of GFP-AD1 and GFP-AD6. This type of localization divergence between N-terminal and C-terminal tags has been reported in the literature. Palmer and Freeman (2004) observed that all of 16 C-terminal fusion proteins localized to cellular compartments in accordance with previous studies and/or bioinformatic predictions, but fewer than half of 16 N-terminal fusions localized correctly. Thornton et al. (2011) reported that human ZnT5vA-GFP was localized to the Golgi, whereas GFP-ZnT5vA was localized to the ER. Reuter et al. (2016) demonstrated the different intracellular localization occurring between *Trichoderma reesei* hydrophobin fusion proteins HFBII-GFP and GFP-HFBII, and between *Fusarium verticillioides* HYD fusion proteins HYD3-GFP and GFP-HYD3, as well as between HYD4-GFP and GFP-HYD4 in transgenic tobacco. Wiemann’s group, after investigating the subcellular localization of more than 100 C- and N-terminal fluorescently tagged human genes, concluded that most signal peptides located at the N-terminus of proteins were masked by the N-terminal GFP fusion. Therefore, they concluded that C-terminal tagging of a protein with GFP was generally superior to N-terminal tagging (Simpson et al., 2000). Our results support their view.

It is also notable that both the N-terminal and C-terminal GFP tags suggested the nuclear localization of AD1, AD6, DODA1, and 5GT (**Figures 2**, **3**), yet none of the proteins was predicted as localized to the nucleus by the predictors used, except perhaps for 5GT, which was forecast a very low probability of nuclear localization by Psort (**Table 2** and Supplementary Table S1). A plausible explanation for this result may be that these proteins do not contain a “regular” nucleus-localization signal (NLS, Fukasawa et al., 2014), and most of the currently available prediction programs rely almost solely on the presence of this kind of signal (Tanz and Small, 2011).

Taken together, the above results and discussion suggest that CYP76AD1 (AD1), CYP76AD6 (AD6), and DODA1 (DOD) are co-localized to the cytoplasm (including the ER) and the nucleus, and that cDOPA5GT (5GT), besides this co-localization, may occur also in the chloroplasts.

Zhou et al. (2007) argued that the subcellular localization of a protein should indicate where it most likely exerts its function. Since CYP76AD1 (AD1), CYP76AD6 (AD6), and DODA1 (DOD), as well as cDOPA5GT, were co-localized to the cytoplasm (including the ER) and the nucleus, and they are recognized as the principal enzymes of the betalain biosynthetic pathway (**Figure 1**), all the reactions that they catalyze may thus take place in the cytoplasm (including the ER) and/or nucleus. Consequently, the final products of these reactions, the betaxanthins and betacyanins (and their intermediates), would be synthesized in the cytoplasm (including the ER) and/or nucleus. This proposition supports, in part, the hypothesis of Trezzini and Zryd (1990) that the formation of *cyclo*-DOPA and its reaction with BA might be cytoplasmic.

## Conclusion

CYP76AD1 (AD1), DODA1 (DOD), and CYP76AD6 (AD6), as well as cDOPA5GT (5GT), from betalain-containing plants are mainly co-localized to the cytoplasm (including the ER) and nucleus in transgenic tobacco leaf cells. Cytoplasmic and nuclear co-localization of these enzymes signifies that betalains and their intermediates are biosynthesized in the cytoplasm and/or in the nucleus of betalain-containing plant cells. This elucidation of the subcellular compartmentation of betalain biosynthesis may promote studies of betalain sorting to vacuole, and should facilitate engineering of the betalain metabolic pathway in both betalain- and non-betalain-containing plants.

## Author Contributions

X-GX, NC, and Z-HY planned and designed the studies. NC and Z-HY performed experiments. NC, Z-HY, and X-GX analyzed the data. NC and X-GX wrote the manuscript.

## Conflict of Interest Statement

The authors declare that the research was conducted in the absence of any commercial or financial relationships that could be construed as a potential conflict of interest.

## Footnotes

1 http://www.cbs.dtu.dk/services/TargetP

2 http://www.cbs.dtu.dk/services/ChloroP

3 http://www.cbs.dtu.dk/services/SignalP/

4 http://aias.biol.uoa.gr/PredSL/

5 http://www.genscript.com/wolf-psort.html

6 http://www.softberry.com

7 http://www.uniprot.org

## Abbreviations

AD1: CYP76AD1
AD5: CYP76AD5
AD6: CYP76AD6
5GT: cDOPA5GT
DOD: DODA1
Gene-GFP: GFP tagged at the C-terminus of the gene
GFP: green fluorescent protein
GFP-Gene: GFP tagged at the N-terminus of the gene
RFP: red fluorescent protein
