# Supplementary material for: Cytosolic and Nuclear Co-localization of Betalain Biosynthetic Enzymes in Tobacco Suggests that Betalains Are Synthesized in the Cytoplasm and/or Nucleus of Betalainic Plant Cells
Source: Front Plant Sci. 2017 May 18;8:831. doi: 10.3389/fpls.2017.00831 (PMC5435750; doi:10.3389/fpls.2017.00831)
Supplement: Supplementary file 1 [file Presentation_1.pdf]

Supplementary Material

Cytosol and nucleus co-localization of betalain biosynthetic enzymes in tobacco suggests the betalains are synthesized in the cytoplasm and/or nucleus of betalain plant cells

Ning Chen, Zhi-Hai Yu, Xing-Guo Xiao*

***: Correspondence**

Xing-Guo Xiao, xiaoxg@cau.edu.cn; xiaoxg06@gmail.com

1 Supplementary Tables

Table S1 Bioinformatic prediction of signal peptide and subcellular localization of CYP76AD1, CYP76AD6, DODA1 and cDOPA5GT

		Cell Localization- Bioinformatic Prediction											Signal Peptide						
Gene name	Length (AA)	WoLF PSORT II _ Over 2		ProtComp 9.0 _ Over 1						Uni Prot	PredSL		TargetP		SignalP			ChloroP	
					LDB	PLDB	NN	PT	ALL	LOC	LOC	CS	LOC	RC	POS	VAL	SIG	cTP	SCR
AD1	497	CYTO	5	PM	0	2.5	0.97	0.25	5.25	MEM	SP	25	SP	1	1-25	0.725	Y	N	0.460
		CHL	4	ER	0	2.5	0	1.8	4.6										
		ER	2																
AD6	499	CHL	7	PM	0	2.4	1	0.14	5.2	MEM	CHL	8	SP	4	1-29	0.407	N	Y	0.527
		CYTO	2	ER	0	2.6	0	1.35	4.6										
DODA1	275	CYTO	8	CYTO	0	5.0	0	2.52	7.08	CYTO	O	U	O	2	1-70	0.147	N	N	0.428
		CYSK	4	CHL	0	0	0	0.07	1.48										
5GT	500	CYTO	7.5	EC	0	0	0.94	0.01	2.31	U	O	U	O	1	1-110	0.184	N	N	0.436
		CYTO_ER	4.5	MIT	0	0	0	2.38	1.03										
		NUC	3	ER	0	0	0	0.85	1.81										
		CYSK	2	PEROX	0	0	0.94	0	2.07										
				CHL	0	0	0	0.25	2.28										

AD1, CYP76AD1; AD6, CYP76AD6; 5GT, cDOPA5GT; AA, amino acids; CHL, chloroplast; CYSK, cytoskeleton; CYTO, cytoplasm; CYTO_ER, both cytoplasm and endoplasmic reticulum; EC, extra cellular; ER, endoplasmic reticulum; MEM, membrane; MIT, mitochondria; NUC, nucleus; PEROX, peroxisome; PM, plasma membrane; LDB, localization from database; PLDB, predicted location from database; NN, neural networks; PT, pentamers; ALL, combined results from LDB, PLDB, NN and PT; LOC, prediction of location; CS, cleavage site; U, unknow; O, any other location (expect cTP, mTP and SP); RC, reliability class; SP, secretory pathway; POS, position; VAL, value; SIG, signal peptide; Y, yes; N, no; cTP, chloroplast transit peptide; mTP, mitochondrial targeting peptide; SCR, score.

2 Supplementary Figures

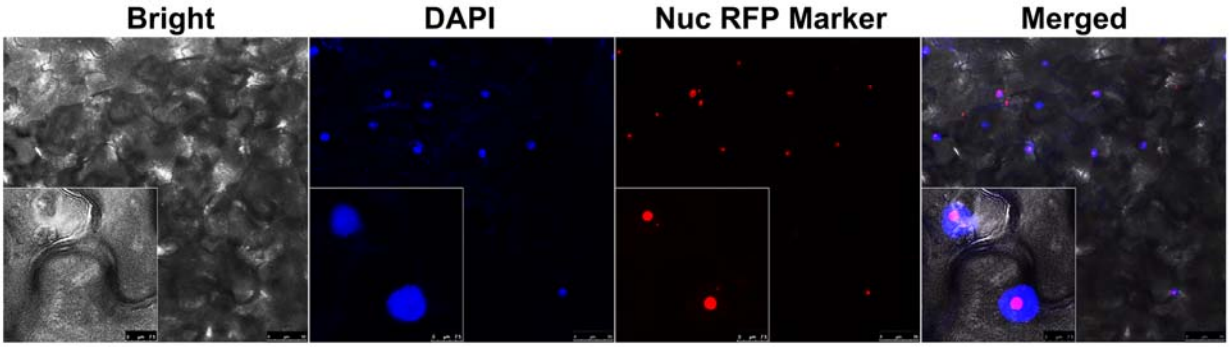

Fig. S1 Confocal fluorescence image of nucleolus marker RFP and DAPI-stained nucleus in transgenic tobacco (*Nicotiana benthamiana*) leaf cells

Insert: amplified image of the nucleus

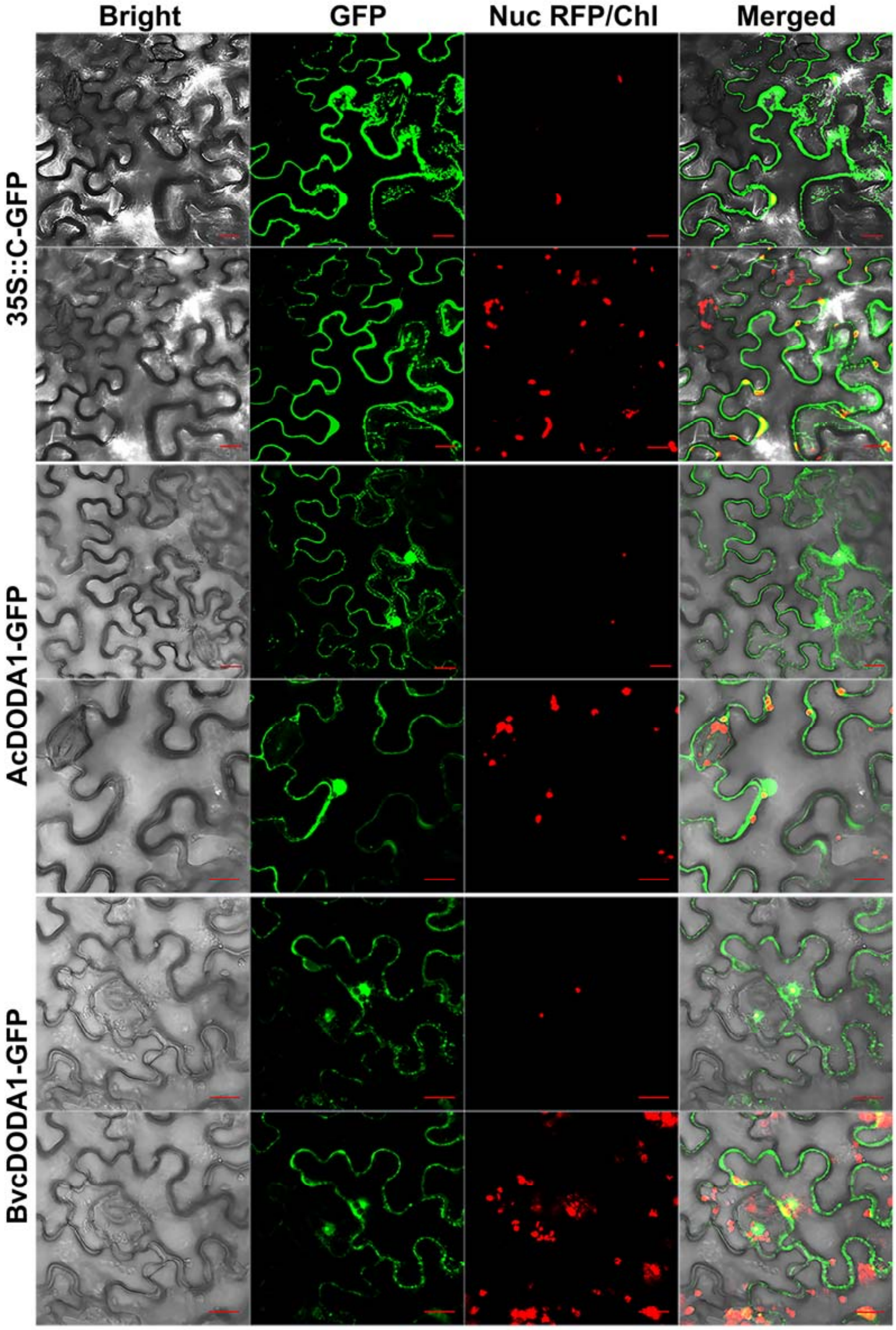

Fig. S2 Confocal GFP fluorescence of AcDODA1-GFP and BvcDODA1-GFP expressed in transgenic tobacco (*Nicotiana benthamiana*) leaf cells

BvcDODA1, DODA1 from red Swiss chard (*Beta vulgaris* subspecies *cicla*); AcDODA1, DODA1 from red amaranth (*Amaranthus cruentus*); Bar=20 μm.
